# Identifying behavior regulatory leverage over mental disorders transcriptomic network hubs toward lifestyle-dependent psychiatric drugs repurposing

**DOI:** 10.1186/s40246-025-00733-w

**Published:** 2025-03-19

**Authors:** Mennatullah Abdelzaher Turky, Ibrahim Youssef, Azza El Amir

**Affiliations:** 1 Faculty of Science, Biotechnology Department, Cairo University, 1 Gamaa Street, Oula, Giza, 12613 Egypt; 2 Faculty of Engineering, Biomedical Engineering Department, Cairo University, Giza, 12613 Egypt; 3 Faculty of Science, Biotechnology Department, Cairo University, Giza, 12613 Egypt

**Keywords:** Mental disorders, Depression, PTSD, Network biology, Machine learning, Drug repurposing, Unhealthy food, Obesity, Smoking, Signal transduction

## Abstract

**Background:**

There is a vast prevalence of mental disorders, but patient responses to psychiatric medication fluctuate. As food choices and daily habits play a fundamental role in this fluctuation, integrating machine learning with network medicine can provide valuable insights into disease systems and the regulatory leverage of lifestyle in mental health.

**Methods:**

This study analyzed coexpression network modules of *MDD* and *PTSD* blood transcriptomic profile using modularity optimization method, the first runner-up of Disease Module Identification *DREAM challenge*. The top disease genes of both MDD and PTSD modules were detected using random forest model. Afterward, the regulatory signature of two predominant habitual phenotypes, diet-induced obesity and smoking, were identified. These transcription/translation regulating factors (*TRFs*) signals were transduced toward the two disorders’ disease genes. A *bipartite network* of drugs that target the TRFS together with PTSD or MDD hubs was constructed.

**Results:**

The research revealed one MDD hub, the CENPJ, which is known to influence intellectual ability. This observation paves the way for additional investigations into the potential of CENPJ as a novel target for MDD therapeutic agents development. Additionally, most of the predicted PTSD hubs were associated with multiple carcinomas, of which the most notable was SHCBP1. SHCBP1 is a known risk factor for glioma, suggesting the importance of continuous monitoring of patients with PTSD to mitigate potential cancer comorbidities. The signaling network illustrated that two PTSD and three MDD biomarkers were co-regulated by habitual phenotype TRFs. 6-Prenylnaringenin and Aflibercept were identified as potential candidates for targeting the MDD and PTSD hubs: ATP6V0A1 and PIGF. However, habitual phenotype TRFs have no leverage over ATP6V0A1 and PIGF.

**Conclusion:**

Combining machine learning and network biology succeeded in revealing biomarkers for two notoriously spreading disorders, MDD and PTSD. This approach offers a non-invasive diagnostic pipeline and identifies potential drug targets that could be repurposed under further investigation. These findings contribute to our understanding of the complex interplay between mental disorders, daily habits, and psychiatric interventions, thereby facilitating more targeted and personalized treatment strategies.

**Supplementary Information:**

The online version contains supplementary material available at 10.1186/s40246-025-00733-w.

## Background

Psychiatric disorders are prominent risk factors for people in different age groups worldwide. [1]. With special attention given to the elderly population, the World Health Organization (WHO) reported that approximately 14% of the elderly population suffers from mental health conditions that develop due to numerous factors, including previous disease history, life events, environmental surroundings, lifestyle, and daily habits [2]. MDD and PTSD have emerged in the population due to several horrifying events, of which COVID-19 is the leading cause [3, 4]. Therefore, there is a dire need for preventive procedures and early diagnosis methods to prevent the adverse effects of MDD and PTSD, the deadliest of which is suicide [1].

However, misdiagnosis of mental disorders opposes global efforts to address mental health conditions. Various studies discuss issues with misleading and biased diagnoses of psychiatric diseases attributable to the complexity of the disorder system, the presence of similar symptoms across multiple disorders, or even incorrect diagnoses by psychiatrists due to patients’ misleading information [5–8].Additionally, misdiagnosis of mental disorders often leads to incorrect psychotic drug prescriptions [9], followed by pivotal consequences such as overmedication, drug side effects, or abuse of the prescribed medication [10, 11]. Consequently, scientific endeavors have intensified to personalize medications, aiming to address improper medication prescription issues in general, and psychiatric drug resistance, in particular [12–15].

One of the strata for approaching individual-tailored medication is nutrition and lifestyle, whereas certain therapeutic approaches require a shift in daily habits and nutrition along with prescribed medications to ensure promising results [16–19]. Nevertheless, creating a meticulous protocol for each individual using traditional behavioral and molecular tests is tedious. Thus, personalized medicine relies on sophisticated strategies, among which network medicine is paramount. Since network medicine works by organizing and merging big data from distinct biological levels (multiple omics layers) in conjunction with social and environmental factors. Subsequently, a network is constructed to aid in the selection of suitable therapies [20–23].

Network medicine has been extensively used to study disease characteristics, ranging from hubs prediction to the multilevel interaction between biological layers of the disease network [24]. One of the most important approaches employed to analyze and detect network modules is the disease module identification challenge of DREAM community. The benchmark challenge was constructed to determine the most effective network analysis algorithms, leading to the identification of the three algorithms that ranked the highest according to the established criteria [25]. Since psychiatric disorders are complex and interconnected [26], network medicine could serve the purpose of discovering the vague network underlaying disorders development, lifestyle correlates, and aid on the development of effective and personalized drugs.

Therefore, multiple studies employed network biology to either uncover molecular complexity of certain mental disorders or lifestyle implications on MDD and PTSD, Some studies investigated the transcriptomic-based network of depression and sleep [27]. Additional research endeavors have sought to identify the association between depression, anxiety, and health-promoting lifestyles by building a psychiatric-scale-based network [28, 29]. The scientific community has attempted to elucidate the molecular mechanisms of MDD utilizing systems biology approaches by compiling previously identified candidate genes into networks or integrating multiple molecular layers. These studies aimed to define the dysregulated biological processes involved in immune dysfunction or the metabolic syndrome prevalent in MDD patients [30, 31].

On the other hand, network analysis of PTSDs’ fMRI provides a better understanding of disorder psychopathology in different brain regions [32]. Multiple attempts have been made to construct and analyze mental health scale-based networks to identify central PTSD symptoms during traumatic life events such as combat experiences and the COVID-19 Pandemic [33, 34]. At the molecular level, the field of network medicine has contributed to revealing the key players in the disorder system. Not only did this field expand to encompass multiple layers, but it also included the identification of potential PTSD hubs in various tissues (reviewed in [35, 36]). Nevertheless, previous research has not used the advantages of machine learning in combination with systems biology approaches to unleash effective and noninvasive diagnostic modules for MDD and PTSD [37, 38]. Moreover, the molecular underpinnings of the relationship between mental health and unhealthy habits are not fully understood. This motivated the rationale of the study pipeline to utilize both the random forest model and co-expression network analysis. Furthermore, the datasets utilized, whether for discovery or validation purposes, were confined to the blood-derived gene expression microarray data. Ultimately, two of the most prevalent detrimental behaviors were selected to investigate how the predominance of poor dietary habits or tobacco consumption elevates the chance of mental well-being deterioration globally [39, 40].

This study aimed to identify MDD and PTSD biomarkers, followed by tracking the causal molecular inference between lifestyle and these disorders. Initially, the pipeline targets the identification of phenotype-specific transcriptomic signatures by integrating statistical-based dataset harmonization. Subsequently, co-expression network analysis and machine-learning model are built to uncover hub genes. The study then examined the transduction of signals from habitual phenotype TRFs into MDD and PTSD biomarkers. In the final stage, a bipartite network connecting candidate genes and drugs is built to yield drug candidates for repurposing in the treatment of patients with MDD and/or PTSD, tailored to their individual lifestyles.

## Methods

### Data harmonization: data filtration, normalization, and batch correction

This study collected blood gene expression raw data from publicly available databases: Gene Expression Omnibus [41],and ArrayExpress [42]. The databases were filtered to include only the microarray series matching the following words (Blood, Whole blood, PBMC, and Peripheral blood) to subtract the results of the expression datasets that only used blood samples. To collect the datasets for obesity, the following words were used: "diet" OR "dietary pattern" OR "food" OR "calorie" OR "eating" OR "eating pattern" OR "BMI" OR "obese" OR "obesity". Search words used to collect the datasets for smoking tobacco were “smoking”, “smoker”, “tobacco”, “smoke”, “smoking behavior”, “cigarette smoking”, and “cigarette”. In addition, the words used to search for the MDD and PTSD datasets were (MDD, major depressive disorder, depression, depressed, and depressive episode) (PTSD, trauma, traumatic, stressful events, stress, and posttraumatic syndrome disorder). The collected data were divided into two parts: discovery and validation. In addition, each dataset encompassed only one of the four phenotypes, in addition to the control cases, and any other phenotype was filtered. The discovery datasets were collected from one platform, whereas the validation datasets were collected from different platforms. All data were normalized in the same manner using the robust multichip average, RMA normalization. *Affy* [43], *Oligo* [44] packages normalized Affymetrix datasets, and *limma* [45] package was used to normalize the Illumina and Agilent datasets. ComBat algorithm from SVA package was used to remove batch effects between the discovery and validation datasets [46], whereas a model was built by considering one of the four phenotypes as a covariate using the generic function model.matrix() in R. Gene Symbols were used to annotate all the probes in both the discovery and validation metadata sets. Meanwhile, the replicated probes for the same gene were summarized by taking the mean probe signal using aggregate() generic function in R. Supplementary Data (Addtional file two) contains the datasets used in this study and their corresponding platforms.

### Identifying blood transcriptomic signatures for MDD, PTSD, obesity, and smoking metadatasets

Differentially expressed genes (DEGs) were identified using a linear regression model [47] implemented through *limma* package. The model was fitted to the data by designing a matrix for the control and test samples. For each of the four phenotypes, the matrix was employed to construct the regression model, and the false discovery rate (FDR) was corrected using the Benjamini–Hochberg method [48] to yield the adjusted p-value. The adjustment was performed using the eBayes() function in limma package. DEGs discovered in each dataset were cross-validated one more time with 2500 iterations, after which the intersection between the discovery and validation cross-validated DEGs was taken. Only positively correlated Log FC values between the two metadata genes were considered for the final differentially expressed gene sets. Ultimately, the gene set for each phenotype was used as the input for further downstream analyses.

### MDD and PTSD transcriptomic signature enrichment

The two disorders were compared with respect to biological processes and immune gene sets that were enriched by the identified DEGs. To enrich the biological processes [49, 50] enrichGO() command from *Clusterprofiler* package [51] were used. In order to relate the terms to their ancestor terms, a tree plot and an emaplot were generated to visualize the enriched terms of the MDD and PTSD DEGs, respectively. Additionally, MsigDB immunologic gene set was downloaded to reveal the enriched gene set identified from the C7 set [52]. Finally, pathway enrichment was performed using three databases: Reactome [53], KEGG [54, 55], and Wikipathways [56]. The results were displayed using a chord and a bar plot generated from the *GOplot* and *ggpubr* R packages, respectively [57, 58].

### Weighted gene co-expression network generation and analysis

A co-expression network was generated with highly correlated gene sets between the discovery and validation DEGs of the two phenotypes of interest: MDD and PTSD. Initially, *psych* package [59] in R was used to generate the similarity matrix using Spearman correlation, as Spearman correlation is less sensitive to outliers [60]. The matrix was filtered only to contain gene pairs with a correlation higher than 0.9 to readily reduce false positives. Analysis was performed using the *MoNET* [61] package of M1 module, the first runner of the Disease Module Identification Dialogue on Reverse Engineering and Assessment (DREAM) Challenge [25]. M1 method involves testing different resolutions to determine the appropriate topological scale of a module [62, 63]. A filtered correlation network was used as the input for M1 module. The network was directed, and the default limits of the clusters were set at 3 and 100, minimum and maximum, respectively.

### Biomarker discovery for MDD and PTSD

The module importance was predicted using a random forest (RF) model [64]. For every permutation, the model randomly split the data to hide 30% of the data, which is an out-of-the-bag sample. This strategy renders RF models immune to overfitting and does not necessarily require training or a test set; thus, RF models have been used for gene prioritization [65]. The RF model was built using *randomForest* package in R [66] with a number of trees (*ntrees*) equal to 50,001. The importance of each gene, sample size of the controls, and phenotypes were calculated. The out-of-the-bag error was retrieved along with the phenotype and the control error, and the randomForestExplainer R package was employed to find the importance frame and calculate the minimum depth frame [67]. Finally, the area under the curve was computed using *PRROC *R package [68] and the receiver operating characteristic (ROC) curve was plotted for the MDD and PTSD RF models.

### Constructing signal transduction network and bipartite network for TRFs and uncovering candidate drug for repurposing

Obesity and smoking signature genes with regulatory functions, whether transcription- or translation-related, were retrieved from MsigDB [52, 69]. A signaling network of the source genes, TRFs, was constructed with the top MDD and PTSD genes as the target nodes. Signor database [70] plugin in Cytoscape 3.10 [71] was used to determine the shortest path between the TRFs of both habitual phenotypes and the top-ranked gene derived from the RF model. This process was repeated for nine transcription factors for the top disease genes built by the model, that is, 10 for MDD and 22 for PTSD. Furthermore, the source signaling genes and signal target genes were retrieved from the Drug–Gene Interaction database (DGIdb), and the repurposing hub was obtained from BROAD Institute [72, 73].

### Statistical analysis

The *p*-value threshold used in this study was set at 0.05. Cross-validation was performed by randomly sampling each metadata point for every phenotype 2500 times. Gene set enrichment analysis (GSEA) was corrected using FDR, and the accepted *p*-value was 0.05. Finally, the random forest model features were identified by a minimum importance of two (*P* < 0.05). All analyses were performed using R version 4.3.1.

## Results

### Datasets preprocessing and harmonization results

**Fig. 1:**
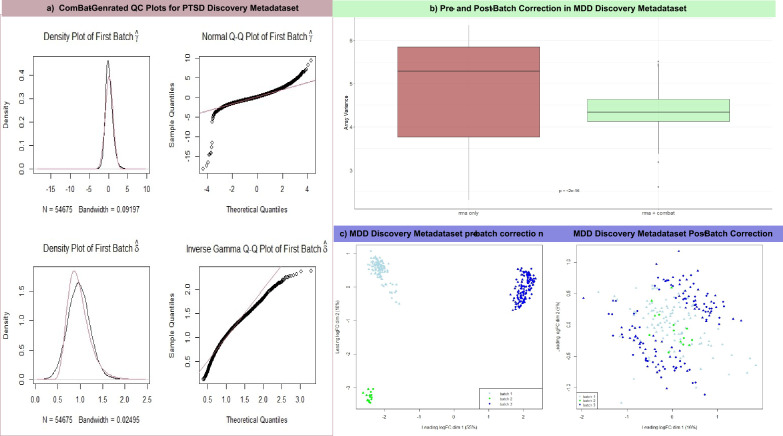
The plots discern the Quality Control (QC) of the harmonization process, **a** shows the density and Q-Q plots generated by *the ComBat* function using the datasets mean and variance on the upper and lower parts, respectively. While **b** shows that the array variance differed significantly before and after batch correction. The last QC stage is the MDS plot in **c**, which illustrates that batch correction was successful for the MDD discovery metadata datasets

This study includes 32 microarray raw series divided into discovery and validation metadata sets, as described in supplementary data T1: MDD metadatasets have 534 samples collected from either Peripheral Blood Mononuclear Cells (PBMC) or peripheral blood of diagnosed MDD patients and 119 control samples collected similarly. The total sample size of the PTSD metadatasets encompasses 273 PTSDs and 320 controls. Additionally, the number of obese and smokers is 209 and 611, respectively. Furthermore, there are 123 controls for obesity and 504 controls for smoking. The preprocessing step involves RMA normalization followed by batch effect removal. In both the discovery and validation metadata, two plots are displayed to prove the validity of the harmonization, and the first plot is the ComBat Quality Check (QC) plots created for PTSD Discovery metadataset (Fig. 1a). The left panel shows the density plots for the batch mean and variance overlap between the kernel estimate (black line) and the parametric estimate (red line) of the empirical batch effect density. The right panel shows that the two lines of parametric and actual ordered batch effects follow similar distributions. Figure 1b is a box plot that tests the variance between MDD discovery metadatast values before and after batch effect removal, indicating a significant reduction in variance (*P* < 2e−16). The final validation graph presents a Multidimensional Scaling (MDS) plot for MDD discovery metadataset before and after batch effect removal, demonstrating the reduction in the first dimension from 55 to 16% following the application of the ComBat function. The validation plots have provided primary confirmation for the subsequent analysis of the four phenotypes under investigation.

### Gene ontology terms, immunologic sets, and pathways enriched by MDD and PTSD transcriptomic signature

**Fig. 2:**
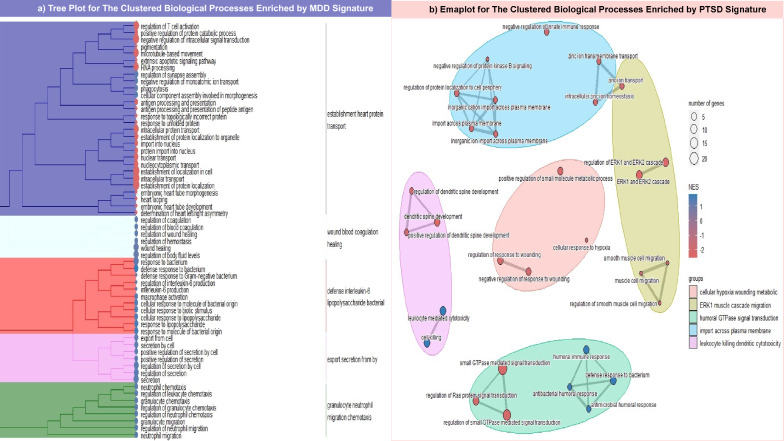
Represents the enriched biological processes of MDD and PTSD DEGs. **a** shows a tree plot for the five ancestral terms enriched by MDD DEGs colored in blue, light-green, red, purple, and green. On the other hand, **b** displays an emaplot for the five ancestral biological terms enriched by PTSD DEGs, the clusters colored uniquely in the following: purple, pink, brown, mint, and light blue. Two scales were found on the right of both plots: the upper scale represents the number of genes detected in each term; the larger the size of the circle, the larger the gene set size of the term, while the lower scale classifies the terms according to its sign the negatively enriched terms found in red, while the positively enriched terms are colored in blue

After normalizing all datasets, the probe sets were annotated with gene symbols and aggregated, yielding 17,312 genes for all the discovery metadatasets. In contrast, the probe sets for each phenotype varied. For example, MDD validation metadataset comprised 6968 genes after aggregation and probe set annotation, whereas the obesity validation metadata set contained 9453 genes. For the smoking and PTSD validation metadata sets, the aggregation and merging processes yielded 8819 and 6535 genes, respectively. Upon harmonization, DEGs associated with MDD and PTSD were evaluated using two criteria: a significant difference (*P* < 0.05) according to the Microarray Quality Control (MAQC) project [74] and a positive correlation between the discovery and validation LogFC values. A total of 352 and 866 DEGs were associated with MDD and PTSD, respectively. The biological process enrichment yielded 68 terms, which were clustered in a tree plot for MDD DEGs. Figure 2a illustrates that three parental terms have positive normalized enrichment scores (NESs). Nevertheless, the cellular positive response secretion term had some downregulated terms, such as regulation of T cell activation and negative regulation of intracellular signal transduction that have NES of 1.54 (*P* = 0.03) and 1.58 (*P* = 0.02), respectively. Moreover, the DEGs related to PTSD (Fig. 2b) are centered around five major terms from a total of 126 terms, two of which are commonly identified in the GSEA of MDD signature. The first common descendant term is defense response to bacterium that has NES equal to 2.30 and 1.79 in PTSD (*P* = 0.004) and MDD (*P* = 0.001), respectively. However, the second term shared between the two analyses has different NESs, as the regulation of response to wounding in the PTSD GSEA exhibited negative enrichment of NES equal to ~ 2.21 (*P* = 0.002), unlike the positive regulation of wound healing in MDD, which had NES equal to ~ 1.75 (*P* = 0.01).

**Fig. 3:**
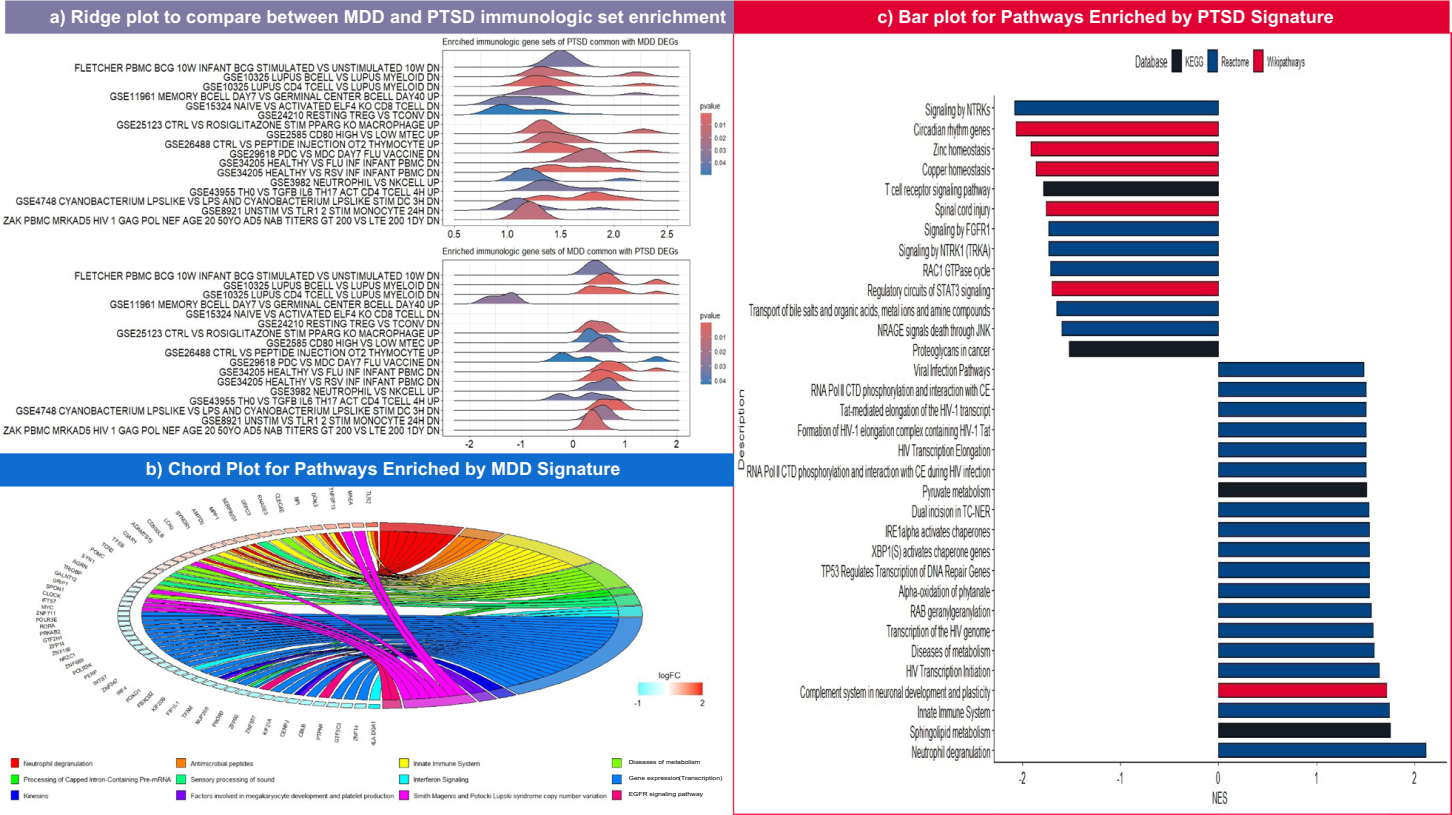
The figure collectively represents a comparison between the MDD and PTSD DEGs in two terms. **a**: compares the commonly enriched C7 gene sets between MDD and PTSD in the form of a ridge plot; the higher the significance of the set, the more it tends to be represented in blue, and vice versa. **b**, **c**: display the cyclic chord and bar plots of enriched pathways of MDD and PTSD DEGs, respectively. The chord color is a code for each enriched pathway, and the square color of each gene represents the LogFC value, whereas red indicates upregulation and cyan indicates downregulation. While the color of each dot in the PTSD bar plot identifies the source database, pathways are ordered from top to bottom in descending order according to the NES value

To investigate DEGs behavior in terms of immunologic gene set enrichment, a ridge plot was generated to compare the NESs of the common terms between PTSD and MDD DEGs (Fig. 3a). Almost all 17 common terms have positive NES except for three gene sets, GSE11961, GSE26488, and GSE43955. While the first one represents genes upregulated in day 7 memory B cells versus day 40 germinal center B cells, it adopts a different pattern: negative enrichment in MDD of NES ranging from 1 to 2 (*P* ~ 0.03), and positive enrichment in PTSD of NES ranging from 1 to 1.5 (*P* = 0.03). The other overlapping gene sets, GSE26488 and GSE43955, are obviously positively enriched in the PTSD DEGs, with a broad peak ranging from 1.2 to 1.6 (*P* < 0.01) and a sharper one at 1.3 (*P* = 0.03), respectively. In contrast, both exhibit an inconsistent pattern in MDD GSEA. Furthermore, the three common pathway databases, Reactome, Wikipathways, and KEGG, were scrutinized for enrichment using the transcriptome signature developed in the present study. In the case of MDD, only 12 pathways were successfully enriched, Fig. 3b shows the presence of three pathways associated with immunogenic reactions involving either innate immune cell activation with NES of 1.87 (*P* = 0.04), antimicrobial peptide formation with NES of 2.05 (*P* = 0.0008), or neutrophil degranulation with NES of 2.6 (*P* = 0.0000977). Three disease pathways were enriched: Smith Magenis and Potocki Lupski syndrome copy number variation, and disease of metabolism with NES of 1.6 (*P* = 0.03), 1.7 (*P* = 0.004), respectively. Surprisingly, the epidermal growth factor receptor (EGFR) pathway and the interferon signaling pathway demonstrate downregulation and scoring the same (NES = − 1.05, 0.03 < *P* < 0.04). For PTSD, 72 pathways were enriched according to the transcriptomic signature (Supplementary Data T2). Figure 3c pinpoints that PTSD transcriptomic signature are positively enriched in viral infection pathways, especially those related to human immunodeficiency virus (HIV) infection which have NES ranging from 1.5 to 1.7 (0.01 < *P* < 0.04). Additionally, three of the fibroblast growth factor receptor (FGFR) protein families were negatively enriched in the neurotrophic tyrosine receptor kinase (NTRK) signaling pathway, all of which has NES of 1.78 (*P* = 0.008). Focusing on homeostasis, zinc and copper homeostasis pathways were found to be negatively enriched with an NES of ~ 1.9 (0.006 > *P* > 0.008), and the transport of bile salts and organic acids, metal ions, and amine compounds pathways were suppressed, with a negative NES of − 1.6 (*P* = 0.01). Some pathways related to neuronal activity, such as circadian rhythm genes and protein–protein interactions at synapses, scored negatively for NES, whereas the scores range between 1.8 and 2 (0.003 < *P* < 0.02). Although MDD and PTSD signatures exhibited enrichment of two pathways: metabolic disease and neutrophil degranulation, the NES was slightly lower in the case of PTSD as metabolic disease enrichment score is 1.58 (*P* = 0.03) and Neutrophil degranulation NES is 2.12 (*P* = 0.0000782).

### Co-expression network analysis and biomarkers detection from the transcriptomic signatures of MDD and PTSD.

**Fig. 4:**
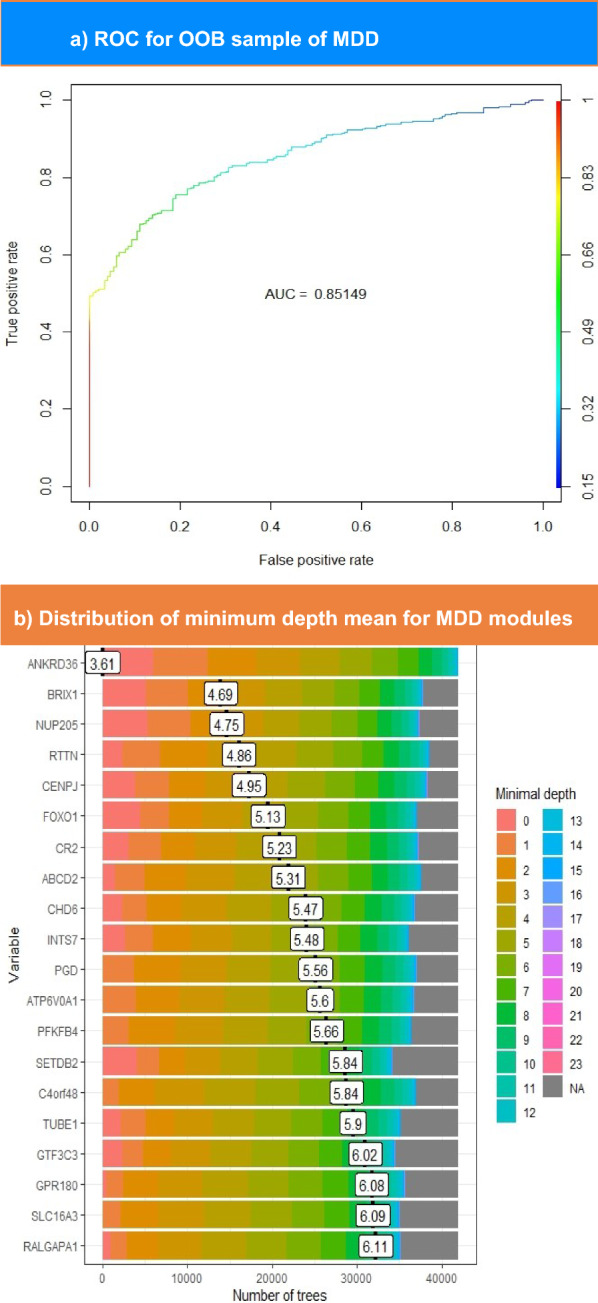
The upper part shows the ROC curve for OOB sampling built for MDD modules. This plot illustrates the performance of the model in distinguishing between patients with MDD and controls. The color scale ranges from blue to red, such that blue indicates the lowest AUC value and red indicates higher values. The lower section represents the distribution of the mean minimum depth for top-ranked MDD genes. The genes with the lowest minimal depth are colored pink, and the color of the scale changes until it reaches gray at the end of the permutations

The correlation matrix built by computing the Spearman correlation between MDD signatures yielded 34 nodes and three communities, whereas the PTSD matrix yielded 592 nodes and 57 communities upon running M1 module analysis. Subsequently, a random forest model was constructed for communities to detect hubs. As shown in Table 1, ten genes passed the significance threshold (*P* < *0.01*) from the MDD signature. Figure 4a shows the ROC curve, which illustrates that the model had an AUC value of 0.85. The distribution of mean minimum depths for MDD top features is displayed in Fig. 4b. This figure illustrates that the top hub ANKRD36 exhibits a mean minimum depth distribution of 3.61.

**Table 1:** Lists the importance of MDD modules in the RF model, which is composed of three columns

Gene symbol	Significance	Importance
ANKRD36	Yes	10.95144
NUP205	Yes	8.793977
BRIX1	Yes	8.507454
FOXO1	Yes	7.451071
CENPJ	Yes	7.038324
SETDB2	No	6.300239
CR2	No	6.131017
RTTN	No	6.010109
INTS7	Yes	5.623512
CHD6	No	5.449391
GTF3C3	No	4.8632
ABCD2	No	4.861166
TUBE1	No	4.784869
RALGAPA1	No	3.798688
PFKFB4	Yes	3.64415
PGD	Yes	3.612938
ATP6V0A1	Yes	3.595073
GPR180	No	3.444351
C4orf48	Yes	3.441046
PTPRK	No	3.257066
HSF2	No	3.227333
TNFSF14	No	3.191718
TST	No	3.145025
SLC16A3	No	3.112939
CBLB	No	3.087692
PAQR3	No	3.04582
CWF19L2	No	3.034433
CEACAM4	No	3.005688
MFN2	No	2.865313
TFEB	No	2.862644
PHC1	No	2.829178
EVI5L	No	2.815048
CLDND1	No	2.681666
UNC93B1	No	2.536419

The gene symbols their corresponding importance values is in the first and last one, respectively. The second one indicates whether each module passed the significance threshold or failed; the default p-value threshold in the model was 0.01

**Fig. 5:**
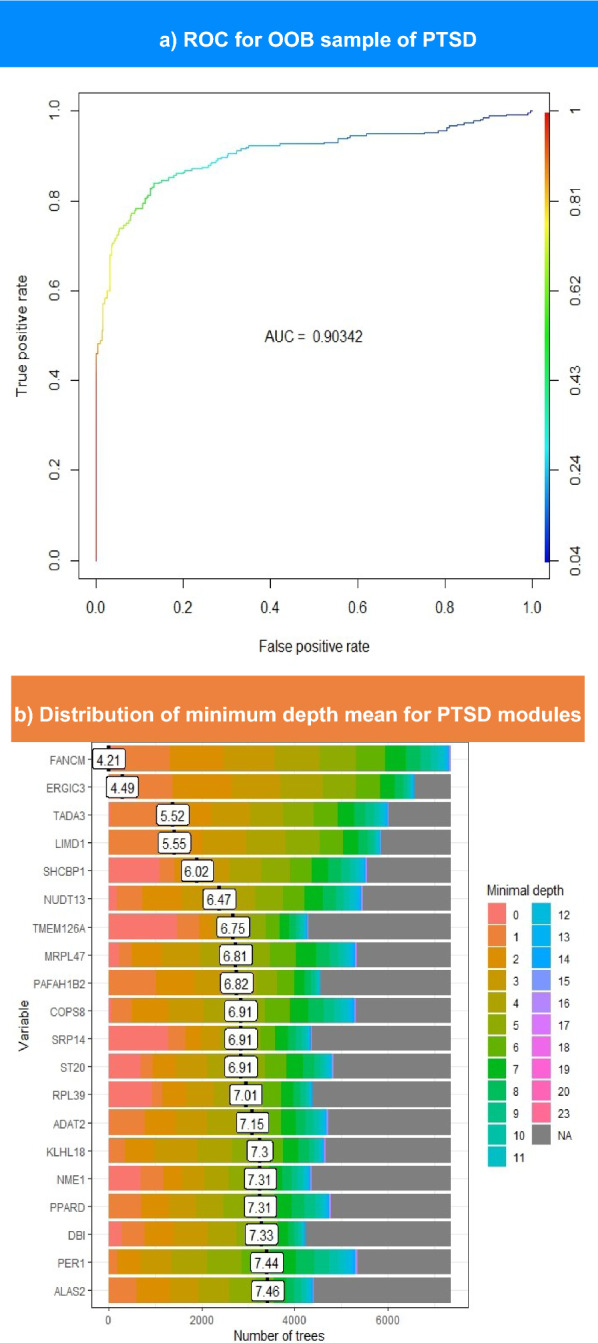
The upper part shows the ROC curve for OOB sampling built for PTSD modules. This plot illustrates the performance of the model in distinguishing between patients with PTSD and controls. The color scale ranges from blue to red, such that blue indicates the lowest AUC value and red indicates higher values. The lower section represents the distribution of the mean minimum depth for top-ranked PTSD genes. The genes with the lowest minimal depth are colored pink, and the color of the scale changes until it reaches gray at the end of the permutations

In the RF model of PTSD modules, 22 genes exceeded an importance value of 2. The ROC curve and minimum depth distribution graph were produced for PTSD hubs, as illustrated in Fig. 5a and b, respectively. However, Fig. 5a shows that PTSD model demonstrates a slightly higher AUC (0.9) compared to MDD model. As presented in Table 2, TMEM126A exhibits the highest importance score of 4.17. Nevertheless, the latter does not own the minimal mean depth, as FANCM records the lowest mean depth of 4.2, while TMEM126A ranks sixth lowest, scoring 6.75 (Fig. 5b). Overall, MDD hubs seem to have higher importance scores than PTSD hubs. In contrast, PTSD mean minimum depth scores is slightly higher than those of MDD hubs, as presented in Tables 1 and 2.

**Table 2:** Lists the importance of PTSD modules in the RF model, which is composed of three columns

Gene symbol	Significance	Importance
TMEM126A	Yes	4.171664721
SRP14	Yes	3.612324079
SHCBP1	Yes	3.200501377
GGCT	Yes	3.182622332
ACAT2	No	3.16227449
CHCHD7	Yes	2.955685014
INPP1	Yes	2.884858403
POLR2G	Yes	2.758969793
RPL39	Yes	2.652354724
GPD1L	Yes	2.590220474
CDC26	Yes	2.565066794
MFAP1	Yes	2.541240161
TTF1	Yes	2.538019041
NDUFAF1	Yes	2.531358526
TBCC	No	2.521683911
EIF2AK4	Yes	2.489983947
ZNF738	Yes	2.359461135
PIGF	Yes	2.142219187
ALG6	Yes	2.088173914
NME1	Yes	2.0754141
ST20	Yes	2.070198423
GYPC	Yes	2.058727396
TAF7	Yes	2.042329917
PIGC	Yes	2.023403725

The gene symbols their corresponding importance values is in the first and last one, respectively. The second one indicates whether each module passed the significance threshold or failed; the default p-value threshold in the model was 0.01

### Exploring the signaling relationship between habits and mental health.

**Fig. 6:**
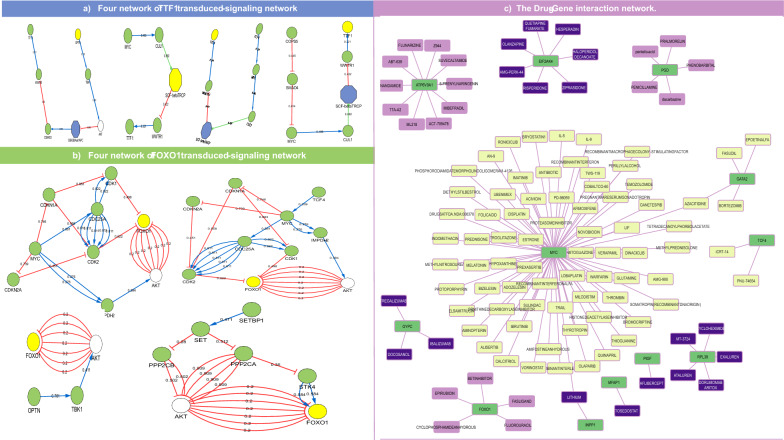
The figure shows the signal transduction network and drug-gene interaction network. **a**, **b** shows the four signal transduction paths of the four TRFs toward TTF1 gene in the upper part and FOXO1 gene in the lower part. The blue line in the network represents positive regulation, and the red-colored edges represent negative regulation of the target node. In **c** the drugs targeting lifestyle-based and disease genes are shown. Drug nodes are colored as follows: purple, dark blue, and faint yellow for drugs targeting MDD, PTSD hubs, and TRFs (MYC, GATA2, TCF4), respectively. In contrast, the gene source nodes are green

To determine the regulatory relationship between habitual phenotypes (diet-induced obesity and smoking) and the two disorder hubs, the same preprocessing procedure was used to construct a transcriptomic signature. In the obesity signature, eight genes were found to possess regulatory functions: COPS5, GATA2, MORF4L1, OPTN, PFDN5, SETBP1, TCF4, and ZBTB16. Moreover, smoking signature included one transcription factor: MYC. The shortest path between the nine regulatory genes and MDD and PTSD disease genes was identified. The PTSD hubs, SHCBP1 and TTF1, were predicted to indirectly receive signals from the regulatory signatures of obesity and smoking. The signal transduction networks associated with the four regulatory factors that target TTF1 are shown in Fig. 6a. In contrast, the MDD hub genes FOXO1, CENPJ, and PGD were hypothesized to have signals transduced from the TRF signatures of the habitual phenotypes. Figure 6b shows the shortest paths of FOXO1 for the four TRFs. Of the nine TRFs, two genes, ZBTB16 and PFDN5, were found to have no connection with any MDD or PTSD hub. Generally, TRFs seemed to transduce shorter signal paths toward PTSD disease genes than MDD. After retrieving the drugs that interacted with either TRFs or hub genes, MYC, the smoking regulatory signature, was predicted to interact with 71 compounds. The Obesity TRFs GATA2 and TCF4 were predicted to interact with four and two interactors, respectively. Azacitidine commonly interacts with GATA2 and MYC. With respect to the drugs retrieved, only FOXO1, PGD, and ATP6V0A1 from the MDD hubs were predicted to have 5, 3, and 10 interactors, respectively. In contrast, 6 of the top 20 PTSD genes were hypothesized to interact with compounds retrieved from DGIdb and BROAD repurposing hub. Notably, one PTSD hub, INPP1, has one common FDA-approved drug with MYC,lithium, as shown in Fig. 6c.

## Discussion

This study began with sample harmonization and the QC plots for the process were repeated for all phenotypes. MDS plots demonstrated expression patterns across different dataset [75], highlighting how MDS illustrates the difference before and after batch correction. After sample harmonization, MDD DEGs positively enriched the regulation of interleukin-6 (IL-6) production, which is consistent with the results of multiple previous studies proving that multiple interleukins are dysregulated in the blood of MDD patients, including IL-6 [76–79]. Moreover, T-cell activation was downregulated, whereas neutrophil chemotaxis was upregulated. The surge in (NLR) in patients with MDD has been discussed as an inflammatory biomarker for depression or as another disease etiology associated with MDD [80–84].

Overall, pathway enrichment of the MDD DEGs supported the biological process results, as shown in Fig. 3b. Moreover, interferon (IFN) signaling was found to be influenced by pathway enrichment. IFN plays a crucial role in the pathogenesis of depression-related inflammation, especially in patients receiving IFN-induced treatment for hepatitis C infection, However, previous research results have been inconsistent. One study either reported that IFN-induced treatment does not necessitate depression development [85] or that IFN blood levels are downregulated [86], and aligned with the GSEA results (Fig. 3b). Thus, personalized check-ups should be conducted before prescribing medications to restore the IFN levels.

Notably, the child terms in the embryonic heart tube asymmetry cluster have negative NESs, consistent with previous association studies investigating the impact of prenatal depression on fetal development and embryonic heart development [87–90]. Thus, this observation entails mentoring infants' heart health by examining the maternal blood of MDD patients.

The enrichment of DEGs identified in PTSD signature indicated that the biological processes involved in dendritic spine development were both impaired and downregulated. This finding is in agreement with those of previous studies [91, 92]. Previous research have investigated the causal mechanism of this impairment upon exposure to chronic stress, and some have concluded that the reason for dendritic spine dysmorphogenesis is the substantial increase in glucocorticoid levels [93, 94]. Pathway enrichment analysis complemented the biological process enrichment analysis, revealing many of the FGFR family members (FGFR1,3, and 4); the NTRK1 signaling pathway was negatively enriched, whereas the dysregulation of both families is vital for the development of cognitive impairment and neural stem cell abnormal differentiation [95–97], which were preliminarily reported in PTSD pathogenesis [98, 99].

Nakajima et al. studied the effect of NTRK1 mutation in mice and reported that the mice exhibited depression-like behavior, suggesting that NTKR1 may serve as a prognostic marker for bipolar and depressive disorders [100]. However, the FGFR system is dysregulated in MDD patients [101]. This link suggests that redirecting treatment for MDD patients with comorbid PTSD could trigger rebalancing of the NTKR and FGFR signaling systems. Furthermore, the impact of PTSD on plasma membrane homeostasis was clear (Fig. 3c). Similarly, zinc and copper homeostasis was downregulated. Previously, zinc homeostasis disruption was suggested to crucially contribute to the development of neurodegenerative disease neuroinflammation, and oxidative stress in the central nervous system has increased [102, 103]. These findings are consistent with the upregulation of several pathways enriched in the PTSD GSEA, which included the alpha oxidation of phytanate and biological oxidation, as displayed in Fig. 3c. Notably, a decreased zinc balance is correlated with various mood disorders, among which the most noticeable was depression [104]. For this purpose, it is advisable to either use zinc rebalancing strategies as one of the treatment paths or repurpose quondam drugs for PTSD treatment, as these agents act on membrane transporter modulation to regulate zinc ions.

Despite the high similarity between MDD and PTSD-enriched immunologic-related gene sets, only PTSD enrichment analysis revealed the negative regulation of the extracellular signal-regulated kinase 1,2 (ERK1/2) cascade in the same cluster of genes involved in the negative regulation of smooth muscle migration. The ERK1/2 cascade fluctuate due to PTSD [105, 106] and is further associated with gluconeogenesis [107], a key player in the development of diabetes mellitus type 2 (T2D) [108–110]. This finding explains why the wound healing cascade is negatively regulated by PTSD signature (Fig. 2b), unlike in MDD signature (Fig. 2a). Subsequently, this study endorses the avoidance of selective serotonin reuptake inhibitor (SSRI) intake in patients with MDD and PTSD comorbidity, since SSRIs can prevent blood coagulation [111–113].

Disease gene identification has been essential in multiple fields, including diagnostic field, drug discovery, and personalized medicine [114, 115]. Forkhead box protein O1 (FOXO1) is among the most important hubs that contributes to the coordination of stress tolerance, cell differentiation, and proliferation [116, 117]. Considerable evidence has proven that the expression of FOXO1 is manipulated by phosphorylation during depression [118, 119]. Previous studies have suggested that 6-phosphogluconate dehydrogenase (PGD) expression is interrupted due to microRNA expression in schizophrenia and bipolar disorder [120] and fluctuates during frontotemporal lobar degeneration (FTLD) [121]. Additionally, centromere protein J (CENPJ) depletion has been strongly associated with reduced brain size as emphasized in primary microcephaly 6 (MCPH6), severe intellectual disability, and abnormal cilia disassembly in adult neural stem cells [122, 123]. In addition to research on the roles of these genes in brain homeostasis and normal neurogenesis, this study showed that FOXO1, CENPJ, and PGD were significantly interrupted in MDD (Table 1), suggesting that these genes are MDD biomarkers.

Eukaryotic translation initiation factor 2 alpha kinase 4 (EIF2AK4), which has been intensively studied as a psychotic drug target, was also detected [124, 125]. Most of the top 22 genes associated with PTSD were mapped to functions related to transcriptional regulation, mRNA posttranscriptional modification, or translation-related proteins. Most of the top 20 genes associated with PTSD were mapped to functions related to transcriptional regulation, post-transcriptional mRNA modification, or translation-related proteins. Among these proteins, src homology collagen (SHC) SH2 domain-binding protein 1 (SHCBP1) was prioritized in the RF model. The nucleoprotein SHCBP1 has been mentioned in multiple studies as a potential prognostic agent for many types of cancers. [126, 127], one of which was glioma [128, 129]. Additionally, excessive phosphatidylinositol glycan C (PIGC) gene expression contributes to hepatocellular carcinoma lethality [130, 131], while a mutation in the PIGC gene is correlated with epilepsy and seizure disorders [132]. Furthermore, thorough investigation of the thyroid transcription factor 1 (TTF1) gene revealed its crucial role in lung adenocarcinoma as either a diagnostic agent or a target for treatment [133–136]. The results from disease gene discovery for PTSD prove the strong correlation between stress and development (Table 2). Lifestyle has been intensively discussed as a driving factor for mental well-being [137–140]. Although the signaling pathways from TRFs to MDD and PTSD hubs are circuitous (Fig. 6a, b), such paths support the previous hypothesis that an unhealthy lifestyle is a risk factor for mental disorders.

Drug repurposing provides an optimal solution for drug discovery, as it eliminates several steps required for the approval of traditional drugs. Various methods have been used to predict the repositioning of candidate compounds. Methods for repurposing differ based on the availability of data related to either disease or drug candidates. To aid such a purpose, deep learning technologies have led the field of computational methods. Neural Matrix Factorization (NMF) has been widely used in repurposing because of its ability to handle sparse data from disease-drug matrices. NMF depends on latent feature factors (drugs and diseases, for example) to drive a relationship between each using Euclidean distance calculation [141, 142]. These properties have encouraged further enhancements to the model such as Neural Matrix Factorization++(NeuMF + +) and Neural Collective Matrix Factorization (NCMF). Although the two models adopted the same strategy as the traditional NMF, the algorithm either depended on auxiliary data representation, as in NeuMF++, or utilized collected data from manifold matrices, such as NCMF. The former uses Multilayer Perceptrons (MLP) with NMF to handle auxiliary data and integrate it into a representation task that allows NeuMF++ to capture nonlinear relationships, while the latter involves the use of Variational Autoencoders (VAE) so that NCMF can handle noisy data. The last example of advancement in NMF is the Additional Neural Matrix Factorization (ANMF) method, which provides the advantage of using two matrices, a drug-drug and disease-disease similarity matrix, to learn from known associations before proceeding to the final step that involves negative sampling [143–145]. Negative sampling is widely used in drug reprocessing tasks as a savior strategy to handle data sparsity, but in turn introduces a huge bias in the representation task. Several models have counteracted the negative sampling bias, such as self-supervised learning (SSL). SSL handles data sparsity through three consecutive strategies: The first is creating variables of the original data matrices in a process called data augmentation; hence, it uses "self" data instead of negative samples. The second one is comparing drug similarities to each other through contrastive learning, the last one being joint training where the two previous steps take place coherently to feed the representation task of drug-disease association [146]. A more specific model was developed to predict drug-target binding affinity, which is Graph-Contrastive Learning for Drug-Target binding Affinity prediction (GraphCL-DTA). The main difference between SSL and GraphCL-DTA is that the latter uses molecular graph semantics during the contrastive learning process and relies on distinguishing the views of the graph upon augmentation [147]. Therefore, GraphCL-DTA is recommended for further analysis of the drug targets identified in the current study.

This study reports that diet-induced obesity and smoking TRFs transduce signals to genes related to stress and normal intellectual skills, drug repositioning could inspire hope in patients with drug-resistant infections. Cures that reduce inflammation, such as nonsteroidal anti-inflammatory drugs (NSAIDs) [148, 149], ABT-639 [150–152], and 6-Prenylnaringenin[153–155] were shown to interact with either lifestyle-related TRFs or lifestyle-affected hubs (Fig. 6c). Thus, we recommend using one of these candidates along with an appropriate treatment protocol, as previous drugs can alleviate physiological inflammation.

Knowing that PTSD is a risk factor for visual disturbances [156, 157], Supplementary Data T3 reported that aflibercept had the highest interaction score with PIGC. These findings demonstrate the strong potential of PIGF as a PTSD biomarker and suggest the use of aflibercept for the treatment of PTSD patients if signs of blurry vision are present [158, 159]. Moreover, research has shown that penicillamine [160, 161] might resolve symptoms of rheumatoid arthritis (RA) in MDD patients, as depression and RA are strongly correlated [162, 163]. Critically, the observed interaction between MDD hub, PGD, and phenobarbital explains why several case studies have reported a decrease in the psychological state of patients with epilepsy upon long-term intake of these drugs [164, 165].

## Conclusion

This study revealed potential biomarkers for MDD and PTSD by analyzing their transcriptomic signatures. By examining the signal transduction of diet-induced obesity and smoking TRFs, this research provides new insights into the effect of these habits on the regulation of MDD and PTSD biomarkers. It is worth mentioning that this study relies on a single omics layer, which may limit the comprehensive understanding of complex cellular systems for these disorders. Nevertheless, this study presents a reliable pipeline, which is a potential resolution for the persistent challenge in biological research that requires larger sample sizes to enhance the reliability of the analysis results. These findings indicate a novel approach for biomarker detection, drug repurposing, and personalized treatment strategies that take into account patients' lifestyle factors and blood transcriptomic profiles.

## Supplementary Information

Additional file 1.

Additional file 2.

Additional file 3.

Additional file 4.

## Data Availability

All the data analyzed during this study are included in this published article [found in its supplementary information Additional file two].

## Abbreviations

TRFs: Transcription-regulating factors
MDD: Major depressive disorder
PTSD: Post-traumatic stress disorder
WHO: World Health Organization
DREAM: Dialogue on reverse engineering and assessment
RMA: Robust multichip average
FDR: False discovery rate
DEGs: Differentially expressed genes
MoNet: Multi-omic network analysis
RF: Random forest
ROC: Receiver operating characteristic
DGidb: Drug-gene interaction database
LogFC: Logarithmic fold change
GSEA: Gene set enrichment analysis
QC: Quality check
UMAP: Uniform manifold approximation and projection
MAQC: MicroAarray quality control
EGFR: Epidermal growth factor receptor
HIV: Human immunodeficiency virus
FGFR: Fibroblast growth factor receptor
NTRK: Neurotrophic tyrosine receptor kinase
PBMC: Peripheral blood mononuclear cell
PCA: Principal component analysis
IL-6: Interleukin-6
NLR: Neutrophil/lymphocyte ratio
IFN: Interferon
ERK: Extracellular signal-regulated kinases
FOXO1: Forkhead box protein O1
BNDF: Brain-derived neurotrophic factor
PGD: 6-Phosphogluconate dehydrogenase
FTLD: Frontotemporal lobar degeneration
CENPJ: Centromere protein J
MCPH6: Primary microcephaly-6
EIF2AK4: Eukaryotic translation initiation factor 2 alpha kinase 4
SHCBP1: Src homology collagen (SHC) SH2 domain-binding protein 1
PIGC: PhosphatidylInositol glycan C
TIFF1: Thyroid transcription factor 1
NMF: Neural matrix factorization
NeuMF++: Neural matrix factorization++
NCMF: Neural collective matrix factorization
MLP: Multilayer perceptrons
VAE: Variational autoencoders
ADMF: Additional neural matrix factorization
SSL: Self-supervised learning
GraphCL-DTA: Graph-contrastive learning for drug-target binding affinity prediction
FDA: Food and drug administration
NSAIDs: Non-steroidal anti-inflammatory drugs
RA: Rheumatoid arthritis
